# Bioprotective Effect of a Bacteriocin-Producing *Lactococcus lactis* Strain Against *Enterococcus faecium* Isolated from Egyptian Tallaga Cheese

**DOI:** 10.3390/antibiotics15010081

**Published:** 2026-01-13

**Authors:** Seila Agún, Olivia Youssef, Sally Ashry, Beatriz Martínez, Lucía Fernández, Ana Rodríguez, Youssef Abdelshahid, Pilar García

**Affiliations:** 1 Instituto de Productos Lácteos de Asturias, The Spanish National Research Council (CSIC), C/Francisco Pintado Fe, 26, 33011 Oviedo, Asturias, Spain; seila.agun@ipla.csic.es (S.A.); olivia.youssef@ipla.csic.es (O.Y.); bmf1@ipla.csic.es (B.M.); lucia.fernandez@ipla.csic.es (L.F.); anarguez@ipla.csic.es (A.R.); 2 Animal Health Research Institute, Alexandria, Agriculture Research Center (AHRI-ARC), 1 El-Shohada Square, Attarin, Alexandria 21517, Egypt; sashrey@ahri.gov.eg

**Keywords:** food safety, dairy industry, Tallaga cheese, *Enterococcus faecium*, bacteriocin, LAB

## Abstract

**Background/Objectives**: Tallaga cheese is an artisanal form of traditional Egyptian soft white Damietta cheese, characterized by high moisture, elevated salinity, and a limited shelf life, which collectively increase its vulnerability to microbial contamination. Typically produced from raw or minimally heated cow or buffalo milk, Tallaga cheese represents a relevant model for studying emerging food safety challenges. **Methods/Results**: This study revealed marked variability among commercial samples and, unexpectedly, a general absence of typical lactic acid bacteria (LAB) such as *Lactococcus* spp. Instead, enterococci, microorganisms increasingly associated with antimicrobial resistance and virulence traits, emerged as the dominant LAB group, with the detection of *Enterococcus faecium* strains posing particular concern for dairy safety. To address these challenges, the antimicrobial potential of isolated LAB was evaluated against *Latilactobacillus sakei* (CECT 906). Twelve bacteriocin-producing strains were identified: ten *Enterococcus faecalis*, one *E. faecium*, and one *Lactococcus lactis*. Enterococci demonstrated robust tolerance to stress conditions, including high salt concentrations, emphasizing their persistence in dairy environments. Given the relevance of controlling resistant and potentially virulent strains such as *E. faecium*, the bioprotective capacity of two bacteriocinogenic *L. lactis* strains (IPLA 1064 and AHRI ST9) was assessed using a laboratory-scale cheese model. Both strains effectively inhibited *E. faecium* AHRI CH4, achieving reductions of 2.6 and 3.6 log units (99.9%). **Conclusions**: These findings underscore the relevance of bacteriocin-producing *L. lactis* as natural biopreservatives to mitigate emerging threats related to antimicrobial-resistant food-borne pathogens in dairy products.

## 1. Introduction

Tallaga cheese is an artisanal fresh Damietta variety made from raw cow milk, buffalo milk, or a mixture of both, without starter cultures. It is a salty cheese, with about 10% NaCl added directly to the milk before calf rennet addition [1,2]. According to Egyptian Standards (ES) 1008-5 from 2005 [3], this cheese must be ripened under refrigeration for at least two months before marketing, and it must be free from any pathogenic microorganisms or their toxins in 25 g. However, its high moisture and the use of raw or slightly heated milk make it highly susceptible to microbial contamination. The lactic acid bacteria (LAB) present in these cheeses are *Leuconostoc mesenteroides*, *Lactococcus garvieae*, *Aerococcus viridans*, *Lactobacillus versmoldensis* (re-classified as *Companilactobacillus versmoldensis*), *Pediococcus inopinatus*, and *Lactococcus lactis* [1]. Frequently, they exhibit high staphylococcal counts (7.1 to 7.6 log10 CFU/g), low total coliform counts (2.1 to 3.8 log10 CFU/g), and high counts of salt-tolerant microbiota (6.4 to 7.1 log10 CFU/g). For instance, samples from cheese marketed in Assiut city (Egypt) confirmed a high incidence of enterococci (84%), total coliforms (40%), fecal coliforms (16%), *Escherichia coli* (12%), and *Staphylococcus aureus* (84%) [4]. Similarly, Tallaga cheese samples from Zagazig city (Egypt) had high values of cell counts for staphylococci and total coliforms (2.6–3.9 and 0–1.5 log/g, respectively) [5]. These findings highlight the general microbial diversity associated with Tallaga cheese and also the presence of bacterial groups (enterococci, staphylococci, and coliforms) that are recognized to harbor antimicrobial resistance (AMR) genes, virulence determinants, and other traits that complicate their control during processing. Among these, *Enterococcus faecium* and *Staphylococcus aureus* are considered high-priority pathogens [6] which possess the capacity to survive harsh environmental conditions, including high salt, low temperature, and acidic pH, conditions characteristic of many cheese varieties.

Bacteriocins synthesized by LAB occur naturally in foods and have a long history of safe use in the dairy industry [7,8]. Specifically, *L. lactis* is able to produce nisin, a bacteriocin largely studied in different food matrices, with a wide antimicrobial spectrum including Gram-positive bacteria such as *Listeria monocytogenes*, *Clostridium* spp., and *S. aureus* [9]. Bacteriocin-producing *L. lactis* strains have been confirmed as biocontrol agents in real cheese-making conditions [10]. Also, they have been successfully used as adjunct cultures in cheeses such as cheddar, cottage cheese, and soft cheeses, where they effectively controlled the growth of *L. monocytogenes* and other microorganisms during cheese manufacture and storage, without negatively affecting the sensory qualities of the final product [11]. In some studies, nisin-producing *L. lactis* strains used as protective cultures not only improved cheese safety but also contributed to aroma formation through enhanced proteolysis and the suppression of undesirable bacteria [12]. In addition to *Lactococcus* strains, certain *Enterococcus* isolates from Egyptian dairy products have also been proposed as safe protective cultures [13]. This strategy is particularly relevant today, as bacteriocins show activity also against resistant, virulent, and biofilm-forming strains.

In this context, the aim of this work was to collect a number of LAB isolates to further design a protective culture suitable for improving the safety and quality of Tallaga cheese. To our knowledge, this represents the first report involving the use of bacteriocin-producing LAB in Tallaga fresh cheese to curtail the multiplication *of E. faecium*.

## 2. Results

### 2.1. Microbiological Analysis of Tallaga Cheese Reveals Enterococci Dominance

The microbiological safety of traditional cheeses produced from raw milk is always an important issue due to the possible presence of pathogenic bacteria, which would remain in the final product. In the case of Tallaga cheese, information regarding microbial contamination remains scarce. This prompted us to carry out a microbiological study of Tallaga cheeses commonly sold in Alexandria by using fifteen cheese and two buffalo milk samples. The isolation and identification of the main groups of bacteria, including LAB, coliforms, enterococci, *S. aureus*, *E. coli*, *Salmonella*, *L. monocytogenes*, molds, and yeast were performed using selective media (Table 1).

Our results showed that the total number of aerobic mesophilic bacteria (TBC) was approximately 10^5^ CFU/g, whereas the estimated counts of total coliforms were between 10^2^ and 10^3^ CFU/g, and no major pathogenic bacteria (*E. coli*, *Salmonella*, *L. monocytogenes*) or molds were detected. Notably, enterococci were the most abundant LAB (6.5 log units). Regarding samples of buffalo milk, all bacteria were identified as enterococci, specifically *E. faecalis* and *E. faecium*. The concerning results obtained regarding *S. aureus* (5.22 ± 1.4 log CFU/g) (Table 1) led us to identify representative colonies via PCR amplification of the 16S rRNA gene, followed by sequencing. None of the selected colonies were identified as *S. aureus*. Instead, eight colonies were confirmed as *Staphylococcus simulans* and the remaining two as *E. faecalis*.

Due to the most abundant bacteria isolated from these products belonging to the genus *Enterococcus*, we proceeded to confirm this result by taking several colonies from the agar plates and carrying out their identification by 16S rRNA sequencing. Twenty-one out of a total of twenty-three isolates (Table 2) were determined to be *Enterococcus* (17 *E. faecalis* and 4 *E. faecium*). In addition, there was only one strain of *L. lactis* subsp. *lactis* and one strain of *Lactiplantibacillus plantarum*. This prevalence of enterococci must be due to the ability of this genus to grow in these specific conditions and to the inhibitory effect of the high salt content of this cheese on other LAB.

### 2.2. Screening Revealed Predominant Bacteriocin Production Among Enterococci

Amongst the LAB isolated in this study, we searched for potential candidates for the design of a protective culture. This involved looking for strains secreting compounds with antimicrobial activity by testing supernatants of the different bacterial cultures for the presence of putative bacteriocins using the indicator strain *L. sakei* ssp. *sakei* CECT906 (Table 2). *L. lactis* IPLA1064, a nisin Z producer, was used as a positive control. For inhibitory activity detection, the neutralized supernatants were obtained from overnight cultures grown at 30–32 °C in LM17 broth. Among the 23 tested isolates, 13 (1 *L. lactis*, 2 *E. faecium* and 10 *E. faecalis*) turned out to be positive and inhibited the growth of the indicator strains. Therefore, these strains may be able to inhibit undesirable bacteria.

**Figure 1:**
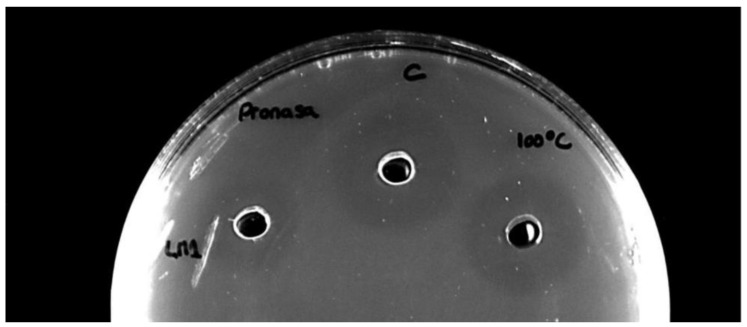
Bacteriocin activity in supernatants of isolated strains determined by using Pronase (final concentration 1 mg/mL, 37 °C, 1 h) and high temperature (100 °C for 15 min) treatment. The supernatant of *E. faecalis* AHRI LM1 was tested using *L. sakei* ssp. *sakei* CECT906 as indicator strain.

Most of the LAB species produce inhibitory extracellular metabolites, mainly organic acids. In order to distinguish them from bacteriocin production, in a subsequent step we checked whether the inhibition detected was resistant to proteolytic attack by pronase and to high temperature treatment. Bacteriocins frequently are resistant to these treatments. Ten *E. faecalis* strains (AHRI CH1, AHRI CH10, AHRI CH11, AHRI CH12, AHRI CH13, AHRI CH15, AHRI CH16, AHRI CH18, AHRI SA2, AHRI LM1), one from *E. faecium* (AHRI CH14), and one from *L. lactis* (AHRI ST9) showed an inhibition halo against the indicator strain (Figure 1).

Then, we proceeded with the detection of bacteriocins secreted by isolates that were able to inhibit *E. faecium* strains also isolated from these cheeses. To do that, the supernatants of all isolates were tested against the whole strain collection from Tallaga cheese. *L. lactis* IPLA1064 was used as a positive control and *L. sakei* CECT906 and *L. cremoris* MG1614 as indicator strains. No inhibitory effects were observed for most of the strains, except *L. lactis* AHRI ST9, whose supernatant inhibited *E. faecium* AHRI CH4 and the indicator strains. The positive control *L. lactis* IPLA1064 also showed an antagonistic effect against *E. faecium* AHRI CH4. Since a LAB strain from cheese (*L. lactis* AHRI ST9) showed inhibitory activity on *E. faecium*, we considered the possibility that it could serve as a protective culture to control the development of this bacterium in cheese.

### 2.3. Antibiotic Resistance and Technological Properties of the Isolated Strains

The characterization of the isolated strains continued by determining their antibiotic resistance profile, following the criteria of EUCAST or CLSI for enterococci and considering the EFSA limits for *E. faecium*. As shown in Table 3, all isolates were sensitive to most antibiotics tested in this study, and only resistance to two different antibiotics (rifampicin and linezolid) was observed. For example, all isolates were inhibited by ampicillin, vancomycin, and linezolid (except for strain AHRI CH12). Inhibition was lower for chloramphenicol and especially for erythromycin. No vancomycin resistance was detected in any strain.

**Figure 2:**
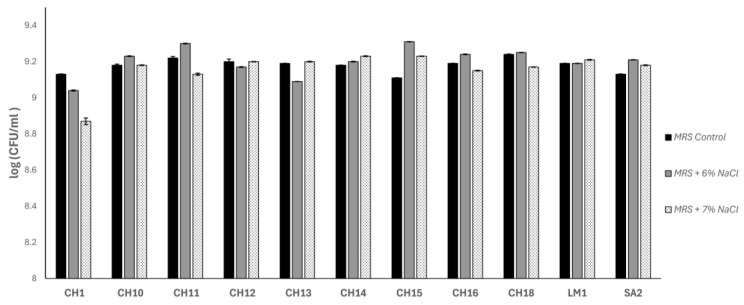
Ability to grow in high salt concentration (NaCl) of selected strains. Data were expressed as mean ± SD (CFU/mL). Black: MRS control, 24 h 30 °C. White dotted: MRS + 6% NaCl, 48 h 30 °C. Gray: MRS + 7% NaCl, 48 h 30 °C. No growth was detected in 8%, 9%, and 10% NaCl.

Additionally, we determined the technological properties of the isolated strains able to synthetize bacteriocin, to evaluate the possibility that some of them could serve as potential protective adjunct cultures. Indeed, a peculiarity of this cheese is the use of milk salting by adding approximately 3 g NaCl per 100 mL of milk (i.e., 3% *w*/*v*) and further coagulation using calf rennet. Since no starter cultures are added, fermentation proceeds via the natural LAB present in the milk. Therefore, the ability to grow at high salt (NaCl) concentrations is advisable. To this end, the isolates’ ability to grow under Tallaga cheese-making conditions was determined. The obtained results indicated that all strains displayed acceptable growth at 6% and 7% NaCl (Figure 2), with no significant differences compared to the control, except for *E. faecalis* AHRI CH9, which was completely inhibited at 7% NaCl. Higher salt concentrations (8–10%) totally avoided bacterial growth for all strains. Overall, their strong tolerance to NaCl should not considered surprising since the strains have been isolated from high salt cheeses.

**Figure 3:**
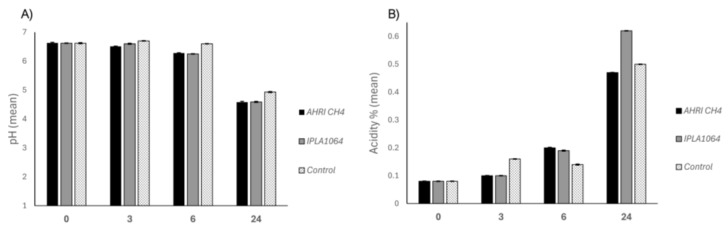
Evolution of pH (**A**) and acidity (**B**) (mean ± SD) of pasteurized milk inoculated with an isolated strain from Tallaga cheese (*E. faecium* AHRI CH4), and the protective strain *L. lactis* IPLA1064. Control milk samples, without any strain, were included (white dotted). Black: *E. faecium* AHRI CH4. Gray: *L. lactis* IPLA1064.

Also important is the ability of the isolated strains to acidify milk. Therefore, we tested the acidity (A%) and pH of inoculated commercial pasteurized milk, both indicative of proper growth in milk (Figure 3). Both strains (*E. faecium* AHRI CH4 and *L. lactis* IPLA1064) were able to grow in milk as they increased the acidity and reduced the pH value below the control-uninoculated milk. We observed an increase in acidity also in the control samples, which could be due to the use of pasteurized, not sterile, milk.

### 2.4. L. lactis Strains Are Protective Cultures Against E. faecium in a Fresh Cheese Model

**Figure 4:**
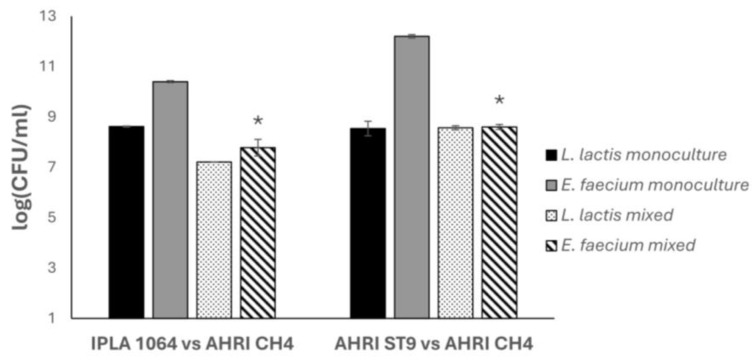
Inhibitory effect of *L. lactis* bacteriocin producers against *E. faecium* AHRI CH4 in lab-scale cheeses. Pasteurized milk was used to prepare three types of cheeses using different cultures: (i) *L. lactis* monoculture, (ii) *E. faecium* monoculture, and (iii) mixed culture. In each case, the appropriate strains were simultaneously inoculated at 10^5^ CFU/mL for *E. faecium* AHRI CH4 and 10^7^ CFU/mL for the protective cultures. (**Left graph**): *L. lactis* IPLA1064 and (**right graph**): *L. lactis* AHRI ST9. Data were obtained after 24 h of incubation at 26 °C. Student’s *t*-test was performed to compare *E. faecium* AHRI CH4 counts in the absence and in the presence of the protective cultures (IPLA1064 or AHRI ST9), * *p* < 0.05.

In this context, we decided to select two strains for the evaluation of their antimicrobial potential as protective strains (*L. lactis* IPLA1064 and *L. lactis* AHRI ST9), both bacteriocin producers, against *E. faecium* AHRI CH4 and confirm their potential using a lab-scale cheese model. The effect of in situ bacteriocinogenic producers on the behavior of *E. faecium* in fresh cheese was determined. Pasteurized whole milk was inoculated with *L. lactis* AHRI ST9 (monoculture), *L. lactis* IPLA1064 (monoculture), *E. faecium* AHRI CH4 (monoculture), and both strains (*L. lactis* plus *E. faecium*) together (mixed culture) at 26 °C and incubated for 24 h. *L. lactis* strains in monoculture grew to reach 8.6 log units. Regarding the mixture cultures, after counting on differential media (Figure 4) the results showed the growth of the protective culture *L. lactis* IPLA1064 to reach 7.21 log units, whereas the *E. faecium* AHRI CH4 growth was reduced from 10.4 to 7.78 log units (reduction of 2.62 log units, 99.98%). Indeed, in the absence of a protective culture, *E. faecium* grew reaching large numbers. Similarly, lab-scale cheeses were performed using *L. lactis* AHRI ST9 as a protective culture against *E. faecium* AHRI CH4 (Figure 4). Whereas the protective culture grew up to 8.57 log units, a reduction of about 3.6 log units (99.97%) was observed for *E. faecium* AHRI CH4 (from 12.2 to 8.6 log CFU/g). Overall, enterococci counts were reduced in all the samples of fresh cheese that had been inoculated with *L. lactis*, regardless of the strain used. Nonetheless, such a reduction was more pronounced in the presence of *L. lactis* AHRI ST9 (Figure 4).

Therefore, our results indicate that manufacturing cheese using a bacteriocin-producing starter not only reinforces the inhibition of *E. faecium* growth but also provides an effective strategy to mitigate the risks posed by antimicrobial-resistant and potentially virulent strains during Tallaga cheese production.

## 3. Materials and Methods

### 3.1. Bacterial Strains and Growth Conditions

Strains used in this study are shown in Table 2, including LAB isolated from Tallaga cheese and buffalo milk, the nisin-producing strain *L. lactis* IPLA1064 [14], and the antimicrobial indicator strains *Latilactobacillus sakei* ssp. *sakei* CECT906 and *Lactococcus cremoris* MG1614 [15]. All LAB strains were routinely grown in M17 (Formedium, Norfolk, UK) supplemented with 0.5% lactose at 30 °C. *L. sakei* ssp. *sakei* was plated on Man-Rogosa-Sharpe (MRS) agar (Difco, Franklin Lakes, NJ, USA) and incubated at 30 °C for 48 h.

### 3.2. Microbiological Analysis of Commercial Egyptian Tallaga Cheeses

Fifteen artisanal Tallaga cheese samples (500 g each) and two buffalo milk samples were collected from various dairy shops in the Alexandria Governorate (Egypt) and transported in iceboxes to the Alexandria Provincial Laboratory (APL-AHRI). A measurement of 25 g of each cheese sample was homogenized in a sterile Stomacher bag with 225 mL of prewarmed 2% sodium citrate (1:10 *w/v* dilution). Decimal dilutions of the resulting homogenates were prepared in quarter strength Ringer solution (Oxoid, Basingstoke, UK) and plated in duplicate on the appropriate medium by pour (1 mL) or spread (0.1 mL) plating. In addition, two buffalo milk samples were analyzed; 25 mL of milk was mixed with 225 mL of saline solution (1:10 *v*/*v*) from which serial dilutions were prepared and 0.1 mL were spread in duplicate on MRS/M17 agar media for detection of lactic acid bacteria. Following the pour-plate method, total aerobic plate counts (TPCs) were enumerated on Plate Count Agar (PCA; Scharlau, Sentmenat, Spain) after incubation at 30 °C for 72 h and coliform group bacteria were determined on Violet Red Bile agar (VRBA; Scharlau, Spain) after incubation at 37 °C for 24 h. Following the spread-plate method, enterococci were enumerated on Kenner Fecal (KF; Scharlau, Spain) agar and incubated at 37 °C for 48 h. LAB group were grown on MRS agar and incubated at 30 °C for 72 h. *S. aureus* counts were determined on Mannitol salt agar (MSA; Titan Biotech Ltd., India) and Baird-Parker agar (BP; AppliChem, Darmstadt, Germany) and incubated at 37 °C for 36 h. Yeast and mold counts were enumerated on Saboraud Dextrose Agar (SDA; Himedia, Bhiwadi, India) after incubation at 21 °C or room temperature for 4 days. Detection of *E. coli*, *Salmonella*, and *L. monocytogenes* was carried out as follows: *E. coli* was assessed both with and without selective enrichment (Buffered Peptone Water, BPW) and subsequently plated on Eosin Methylene Blue (EMB) agar (Maharashtra, Himedia, India) after incubation at 37 °C for 24 h. *Salmonella* was similarly analyzed with and without enrichment in Rappaport-Vassiliadis (RVS) medium (Oxoid, Basingstoke, UK), and detected on Xylose Lysine Deoxycholate (XLD) agar (Oxoid, Basingstoke, UK) following incubation at 37 °C for 24 h. *L. monocytogenes* was identified both with and without enrichment in buffered Listeria enrichment broth (BLEB) (Biolife, Milano, Italy), and then detected on Listeria Oxford agar (Biolife, Milano, Italy) and incubated at 35 °C for 48 h. All analyses were performed following the protocols outlined in the Bacteriological Analytical Manual (BAM) of the U.S. Food and Drug Administration (FDA, 1998) [16]. For identification of the isolated strains, PCR amplification of the 16S rRNA gene was performed [17]. To do that, genomic DNA was extracted using the GenElute Bacterial Genomic DNA Kit (Sigma, Madrid, Spain), according to the manufacturer’s instructions, with an additional lysis step using lysozyme (5 mg/mL) to ensure efficient disruption of Gram-positive bacteria.

### 3.3. Antimicrobial Potential of LAB Isolated from Tallaga Cheese

Strain characterization for antimicrobial production was performed using overnight cultures grown in LM17 broth, which were centrifuged at 12,000 rpm for 10 min at 4 °C. The resulting supernatants were neutralized to pH 7.0 ± 0.2 with 1 M NaOH, and sterilized by filtration through 0.22 µm membrane filters (VWR, Llinars del Vallès, Spain) to obtain cell-free supernatants (CFS). Antimicrobial activity assays of CFS were determined by the agar-well diffusion method (lawn and spot test) [18] by using *L. sakei* ssp. *sakei* CECT906 as the indicator strain, and the nisin-producing strain *L. lactis* IPLA1064 as positive control [14]. In order to confirm the presence of bacteriocins in the supernatants, aliquots were subjected to specific treatments using heat and proteases. CFS were exposed to 100 °C for 15 min. For enzymatic digestion, a Pronase solution (Sigma, St. Louis, MO, USA) (1 mg/mL in 50 mM phosphate buffer, pH 7.0) was prepared and mixed in equal volumes with the heat-treated CFS. The mixtures were incubated at 37 °C for 1 h and then the antimicrobial activity was tested against the indicator strains (see above).

### 3.4. Evaluation of the Technological Properties of LAB Strains

The ability to grow in the presence of a high NaCl concentration was tested by obtaining overnight cultures of each strain grown in MRS medium at 30 °C for 18 h. These cultures (at 1%, *v*/*v*) were used to inoculate 5 mL of MRS supplemented with increasing NaCl concentrations (0%, 2%, 4%, 6.5%, and 10% *w*/*v*). Cultures were incubated in the same conditions for 24, 48, and 72 h. Growth was recorded by counting the CFU/mL on MRS plates and compared to the control culture grown without NaCl. The assays were performed in triplicate. In addition, LAB strains were tested for their ability to grow and acidify the milk. Titratable acidity and pH of commercial pasteurized milk (CPSM; Labanita, Alexandria, Egypt) inoculated with the tested strains were determined at 3, 6, and 24 h of incubation at 30 °C. A measurement of 10 mL of fermented milk was mixed with 0.111 N NaOH (Dornic solution) under continuous stirring until the pH reached 8.2. Results were expressed as grams of lactic acid per 100 mL of milk culture. pH measurements were performed using a calibrated pH meter (micropH2001; Crison, Alella, Spain).

### 3.5. Determination of the Antibiotic Resistance Profiles of the Strains

The antimicrobial susceptibility of the isolates was evaluated by using disks containing the following antibiotics and concentrations (µg/disk): Ampicillin 10; Vancomycin 5; Gentamicin 10; Streptomycin 10; Erythromycin 15; Chloramphenicol 30; Tobramycin 10; Rifampicin 5; Linezolid 30; Oxacillin 5. The assay was performed by the Kirby–Bauer disk diffusion method after strains were activated on MRS agar. A culture of 1.5 × 10^8^ CFU/mL is spread uniformly across the surface of the Mueller–Hinton agar (MHA) plate (Oxoid, Basingstoke, UK) with a sterile cotton swab. Then, antibiotic disks were placed by means of dispenser. After incubation (24 h, 30 °C), bacterial strains were evaluated as resistant, intermedium-grade, and susceptible according to the criteria of the CLSI [19] by measuring inhibition zone diameters around the antibiotic disks.

### 3.6. Use of Protective Strains to Control Contaminants’ Development in a Cheese Model

The assay was performed as previously described with some changes [20]. Briefly, cheeses were prepared in 15 mL polypropylene tubes by mixing 2 mL of pasteurized whole milk (Hacendado, Asturias, Spain), CaCl_2_ (final concentration 0.02%), NaCl (final concentration 0.2%), and commercial liquid rennet (activity 1:1000) (Cuajos Nievi, Bizkaia, Spain). The milk was inoculated with 10^7^ CFU/mL of *L. lactis* IPLA1064 or *L. lactis* AHRI ST9. Milk was also contaminated with 10^6^ CFU/mL of *E. faecium* AHRI CH4. Untreated control samples were included in all experiments. Samples were incubated at 26 °C for 24 h. Following incubation, samples were homogenized by vortexing and the number of viable cells present in the curd was determined by serial dilution in PBS and subsequent spread on LM17 (for *Lactococcus*) and KF (for *Enterococcus*) plates, which were incubated at 32 °C and 37 °C for 24 h, respectively.

### 3.7. Statistical Analysis

All the assays were carried out with three independent biological replicates. These data were analyzed with Student’s *t*-test and *p*-values < 0.05 were considered significant. Two samples in which bacterial counts were below the detectable limit were assigned a value of 100 CFU/mL (the detection limit) for the purposes of statistical analysis and graphical representation.

## 4. Discussion

Recent studies have raised microbiological safety concerns regarding traditional Egyptian cheeses made from raw milk, particularly due to the frequent detection of pathogenic bacteria and the increasing prevalence of virulence and antibiotic resistance determinants [21,22,23]. Indeed, high diversity in microbial communities was recently confirmed with samples of raw or fermented milk and cheeses collected from traditional cheese-making factories, local markets, and farmhouses located in the Delta area of Egypt [24]. Cheeses such as Ras frequently exceed permissible levels of coliforms, yeasts, molds, and anaerobic spore-forming bacteria (*Clostridium perfringens*), with the occasional detection of methicillin-resistant *S. aureus* [22]. Pathogens like *L. monocytogenes*, *Salmonella* spp., and *E. coli* O157:H7 are generally absent. Likewise, Karish cheese, particularly from artisanal production, often shows elevated counts of *S. aureus*, coliforms, *E. coli*, and fungal contaminants [21]. Tallaga cheese is widely consumed, and also especially vulnerable to microbial hazards [25]. However, microbiological analysis of the cheese samples studied in this work showed TBC values indicative of good microbiological quality. In artisanal or traditionally marketed cheeses, particularly those made from raw milk or produced with limited regulatory oversight, TBC levels may surpass 10^7^ CFU/g, raising food safety concerns [26]. The estimated counts of total coliforms, between 10^2^ and 10^3^ CFU/g, can also be considered acceptable. Importantly, no major pathogenic bacteria (*E. coli*, *Salmonella*, *L. monocytogenes*) or molds were detected. However, yeast counts over 10^5^ CFU/g are typically deemed unacceptable, as they may be associated with unwanted fermentation, swelling, sour taste, or visible spoilage [27]. Also, there was a high number of colonies in the media used for selecting *S. aureus* (5.22 log units). Due to some Egyptian cheeses (Tallaga, Domiati, Cheddar, and Ras cheeses) having been described as vehicles for the transmission of *S. aureus* carrying antibiotic resistance genes [25], we proceeded to analyze some colonies, which were confirmed as *S. simulans.* This bacterium is occasionally found in cheese, particularly in raw milk varieties, and might be a potential source of foodborne illness because some strains can produce enterotoxins [28].

Regarding the predominant bacteria, enterococci turned out to be the most abundant. Of note, the species *E. faecalis* and *E. faecium* can grow in the presence of NaCl concentrations up to 6.5% (*w*/*v*) [29], which would explain their high prevalence in a cheese in which the manufacturing process involves the addition of large amounts of salt. Similarly to our findings, *Enterococcus durans* and *E. faecalis* were previously reported as dominant species in Turkish white cheese [30]. Regarding *E. faecalis*, there is evidence for participating in cheese ripening in many traditional raw milk cheeses [31]. These bacteria contribute to the development of flavor and aroma through proteolytic and lipolytic activities, and are often dominant during the early stages of cheese ripening. High levels of *E. faecium* have been consistently isolated from traditional Spanish cheeses like Manchego and Idiazábal, as well as Mediterranean cheese varieties such as Greek Feta and Italian Pecorino [32,33,34,35]. Their use in dairy processing remains controversial due to their ability to carry virulence traits and antibiotic resistance genes [36,37]. Their frequent isolation from traditional Egyptian dairy products further highlights the need to balance technological benefits with food safety considerations [24]. It is important to note that all enterococci isolated from Tallaga cheese were sensitive to most antibiotics tested in this study. Remarkably, rifampicin resistance was fairly common in enterococci because it can arise from simple point mutations in the RNA polymerase, which can quickly render resistance [38]. No vancomycin resistance was detected, which is significant due to the emergence of resistance to this antibiotic in *Enterococcus* strains [39].

Although the sampling carried out in this work did not show a worrying microbiological profile, the abundance of *E. faecium* led us to propose an improvement strategy. The use of lactic acid bacteria (LAB) as starter or protective cultures is a promising approach, as these microorganisms can inhibit spoilage and pathogenic bacteria through the production of organic acids, bacteriocins, and other antimicrobial compounds [7]. For cheeses like Tallaga, which are produced without added starters or pasteurization, the introduction of carefully selected autochthonous LAB could provide an effective means to improve safety and consistency while preserving artisanal identity. Among LAB, *L. lactis* is particularly attractive because of its GRAS (generally recognized as safe) or QPS (qualified presumption of safety) status and its ability to produce bacteriocins with well-documented antimicrobial activity [40]. Their application is especially relevant for controlling emerging threats such as antimicrobial-resistant *E. faecium*, which presents additional risks due to its virulence traits and capacity for biofilm formation. Among the isolates characterized in this work, we selected a *L. lactis* AHRI ST9 strain synthetizing a bacteriocin against *E. faecium* AHRI CH4. Several enterococci isolated from this cheese also produce bacteriocins, as deduced from the presence of a halo against indicator strains, but only *L. lactis* AHRI ST9 was able to inhibit *E. faecium*. The evaluation of the antimicrobial potential as a protective strain against *E. faecium* AHRI CH4 was confirmed using a lab-scale cheese model. This allowed showing the reduction in enterococci growth in the presence of lactococci during the coagulation of the milk. This indicates that bacteriocin is secreted from during *L. lactis* AHRI ST9 growth and its activity is effective against *E. faecium* developing in this food matrix.

The use of bacteriocin-producing LAB as protective cultures therefore represents a promising strategy to enhance the microbiological safety of Tallaga cheese. This approach aligns with global trends promoting natural preservation methods and supports efforts to prevent contamination by resistant or virulent pathogens throughout the dairy production chain [24,41,42]. Importantly, implementing such strategies can help maintain the cultural and sensory integrity of artisanal cheeses while addressing critical food safety challenges.

## 5. Conclusions

The microbial profile of traditional Tallaga cheese differs significantly from those typically found in cheeses from other regions, which are usually dominated by *Lactococcus* and *Lactobacillus*. Notably, most of the strains were identified as *E. faecalis*, exhibiting antimicrobial activity and no detected antibiotic resistance, highlighting their role as a key microbial component of Tallaga cheese. In the present study, we sought to identify additional lactic acid bacteria (LAB) capable of producing bacteriocin-like compounds with the potential for food biopreservation, aiming to suppress undesirable or competing microorganisms, including those with antimicrobial resistance or virulence traits. Importantly, we identified a *L. lactis* strain with antagonistic activity against *E. faecium* in a laboratory-scale cheese model. This could represent the potential to control a species of growing concern due to its resistance, virulence, and potential for biofilm formation. Taken together, these results suggest that locally sourced, environmentally adapted strains may serve as protective cultures in cheese production to help improve microbiological safety, control emerging pathogens, and extend shelf life. Future work is needed to confirm their effectiveness in real production settings, with the aim of supporting traditional practices while addressing issues like antimicrobial resistance and pathogen persistence.

## Figures and Tables

**Table 1:** Bacteriological examination of marketed Tallaga cheese (15 samples). Lactic acid bacteria (LAB), coliforms, enterococci, *S. aureus*, *E. coli*, *Salmonella*, *L. monocytogenes*, mold, and yeast are presented as mean and standard deviation (SD) (Log CFU/g). -, negative result.

Bacterial Group	Mean ± SD (Log CFU/g)
Total bacterial count	5.55 ± 1.4
Coliforms	2.7 ± 0.6
Enterococci	6.51 ± 0.5
LAB	6.51 ± 0.5
*S. aureus*	5.22 ± 1.4
*E. coli*	-
*Salmonella*	-
*L. monocytogenes*	-
Molds	-
Yeasts	5.31 ± 1.0

**Table 2:** Bacterial strains isolated from Tallaga cheese and buffalo milk on MRS and LM17 media. Colonies were identified by PCR amplification of 16S rRNA gene and sequencing. The antimicrobial activity of supernatants was determined by using *L. sakei* ssp. *sakei* CECT906 as indicator. ++, strong antimicrobial activity; +, moderate antimicrobial activity; -, no antimicrobial activity.

Code	Source	Bacterial Strains	Antimicrobial Activity
AHRI CH1	Tallaga cheese	*E. faecalis*	+
AHRI CH2	Tallaga cheese	*E. faecium*	-
AHRI CH4	Tallaga cheese	*E. faecium*	++
AHRI CH8	Tallaga cheese	*L. plantarum*	-
AHRI CH9	Tallaga cheese	*E. faecalis*	-
AHRI CH10	Tallaga cheese	*E. faecalis*	+
AHRI CH11	Tallaga cheese	*E. faecalis*	+
AHRI CH12	Tallaga cheese	*E. faecalis*	+
AHRI CH13	Tallaga cheese	*E. faecalis*	+
AHRI CH14	Tallaga cheese	*E. faecium*	+
AHRI CH15	Tallaga cheese	*E. faecalis*	+
AHRI CH16	Tallaga cheese	*E. faecalis*	+
AHRI CH17	Tallaga cheese	*E. faecalis*	-
AHRI CH18	Tallaga cheese	*E. faecalis*	+
AHRI CH19	Tallaga cheese	*E. faecalis*	-
AHRI SA1	Tallaga cheese	*E. faecalis*	-
AHRI SA2	Tallaga cheese	*E. faecalis*	+
AHRI E2	Tallaga cheese	*E. faecium*	-
AHRI LM1	Tallaga cheese	*E. faecalis*	++
AHRI LM2	Tallaga cheese	*E. faecalis*	-
AHRI M4	Buffalo milk	*E. faecalis*	-
AHRI M2	Buffalo milk	*E. faecalis*	-
AHRI ST9	Tallaga cheese	*L. lactis*	+
IPLA1064	IPLA collection	*L. lactis*	+
MG1614	IPLA collection	*L. cremoris*	-
CECT 906	IPLA collection	*L. sakei* ssp. *sakei*	-

**Table 3:** Antibiotic resistance profiles of isolated strains. R: resistant, I: intermediate resistance, S: sensitivity.

Code	Bacterial Strains	Eri	Cl	Rif	Lz	Van	Amp
AHRI CH1	*E. faecalis*	I	S	I	S	S	S
AHRI CH2	*E. faecium*	I	S	R	S	S	S
AHRI CH4	*E. faecium*	I	S	R	S	S	S
AHRI CH9	*E. faecalis*	I	S	R	S	S	S
AHRI CH10	*E. faecalis*	I	S	R	S	S	S
AHRI CH11	*E. faecalis*	I	I	R	S	S	S
AHRI CH12	*E. faecalis*	I	I	R	R	S	S
AHRI CH13	*E. faecalis*	I	S	I	S	S	S
AHRI CH14	*E. faecium*	I	I	R	S	S	S
AHRI CH15	*E. faecalis*	I	I	R	S	S	S
AHRI CH16	*E. faecalis*	I	I	R	S	S	S
AHRI CH18	*E. faecalis*	I	S	R	S	S	S
AHRI SA2	*E. faecalis*	I	I	I	S	S	S
AHRI E2	*E. faecium*	I	S	R	S	S	S
AHRI LM1	*E. faecalis*	I	I	I	S	S	S
AHRI LM2	*E. faecalis*	I	S	I	S	S	S

## Data Availability

The data presented in this study are available on request from the corresponding authors.
